# Cognitive and brain volume correlates of financial literacy assessment in Greek persons with amnestic Mild Cognitive Impairment: a preliminary study

**DOI:** 10.3389/frdem.2026.1730124

**Published:** 2026-04-29

**Authors:** Vaitsa Giannouli

**Affiliations:** Department of Psychology, Democritus University of Thrace, Didymoteicho, Greece

**Keywords:** aMCI, brain volumes, financial literacy, healthy, older adults

## Abstract

**Background:**

Financial literacy is a crucial skill for the aging population. However, so far we have no data coming from Greece regarding financial literacy and cognitive and brain correlates in healthy older adults and amnestic Mild Cognitive Impairment (aMCI) patients.

**Methods:**

In this preliminary study, financial literacy was examined with the Big Three Questions in 15 individuals with aMCI and 15 matched healthy controls. Simultaneous measurements of different regional brain volumes were derived from structural MRI and the cognitive performance was assessed with a number of standardized neuropsychological tests (Rey auditory verbal learning test, clock drawing test trail-making test parts A and B, Boston Naming Test, digit span memory test forward and backward, and verbal fluency task).

**Results:**

For the first time, findings suggest lower financial literacy in aMCI compared to matched controls. Spearman correlations revealed that financial literacy correlates with a number of brain volumes (white matter, gray matter, left amygdala, and right frontal medial cortex). Among the cognitive tests, correlations were found only with trail making parts A and B time to completion.

**Conclusion:**

The above findings are of importance for clinicians as indicators of need for comprehensive financial capacity assessment of older patients susceptible to financial exploitation.

## Introduction

In cross-cultural financial literacy research, the Big Three Questions are widely used in different populations as they can assess an individual’s knowledge of financial concepts for financial decision-making based on information regarding (a) compound interest, (b) inflation, and (c) risk diversification (Lusardi and Mitchell, 2008; Lusardi and Mitchell, 2011). These concepts and their applications are of extreme importance. They can provide us with vital information that can differentiate individuals in a fast and simple way that is relevant to assessing how making daily financial decisions are made (Lusardi and Mitchell, 2011).

Despite the huge implications that performance in such tasks can have for the aging population (healthy older adults as well as older patients), so far a small body of comparative research exists in Greece showing preliminary findings regarding gender differences that favor men, but with no focus on older adults (Petrakis and Panos, 2025). The research in the United States (U.S.) in Mild Cognitive Impairment (MCI) showed that patients with MCI exhibit poorer financial literacy, which is predicted best by semantic memory performance in neuropsychological testing with education mitigating this effect (Han et al., 2015), and brain imaging findings corroborate that greater financial literacy is associated with higher diffusion anisotropy in white matter of non-demented older adults (Han et al., 2016).

Although financial literacy is different from financial capacity as the former is the crystallized knowledge and understanding of financial concepts, while the latter is the ability to apply that knowledge in real-life situations. The Big Three Questions approach to measurement involves both constructs. Therefore, numerous researchers have tried to elucidate the brain underpinnings of financial capacity and financial literacy. More specifically, brain regions of interest for volumetric studies are the angular gyrus (Griffith et al., 2010; Giannouli et al., 2018) and limbic structures such as amygdala (Benavides-Varela et al., 2020; Giannouli et al., 2018) in MCI patients.

Furthermore, in older adults higher financial literacy has been found to correlate with increased functional connectivity between the posterior cingulate cortex (PCC) and other medial brain regions such as the ventromedial prefrontal cortex (vmPFC) (Han et al., 2014). Findings from younger adults corroborate that voxel-based morphometry (VBM) can reveal a positive correlation between financial extravagance (excessive spending of money) and gray matter volume in the caudate nucleus (Yokoyama et al., 2014). While another study revealed positive correlations between the degree of individual behavioral loss aversion and gray matter volume in the superior frontal gyrus (Li et al., 2020) and correlations between brain areas such as the left and right medial frontal cortices and self-reported delay of gratification for money choices in MCI patients (Giannouli, 2025).

In middle-aged and older adults, findings indicate that both structural integrity and functional connectivity within brain regions supporting arithmetic retrieval are positively related to financial ability. Language skills mediate the association between left inferior frontal gyrus (IFG) structural integrity and financial performance. Conversely, connectivity patterns reflecting greater reliance on quantity-based processing, such as calculation, are linked to poorer financial ability (Suárez-Pellicini and McDonough, 2025). Despite the above, financial literacy and the association with the brain and neuropsychological tasks might differ in different populations as voxel-based morphometry correlates of overall financial literacy in older patients can depend on the educational, cultural, and financial context for the older population (Calia et al., 2025).

The primary aim of this preliminary study is to inform more culturally sensitive neuropsychological research and to investigate what are the cognitive and brain volume correlates of financial literacy (using the Big Three Questions). Given the rapidly growing numbers of amnestic MCI (aMCI) patients in Greece (Exarchos et al., 2025), this study will compare for the first time their performance to that of healthy controls.

## Methods

A group of 30 older adults were assessed at the Papanikolaou Hospital, Greece. Fifteen were patients (*n* = 10 women; *n* = 5 men) with a diagnosis of aMCI without depression, and were examined with the Greek standardized version of Mini Mental State Examination (MMSE), which is a screening test of general cognition (Fountoulakis et al., 2000). In addition, the Geriatric Depression Scale (GDS-15), which is a self-report measure of depression in older adults was administered (Fountoulakis et al., 1999). Fifteen healthy volunteers (*n* = 10 women; *n* = 5 men) were also examined with the same screening tests. The criteria for the diagnosis of aMCI were those used in a previous study (Giannouli and Tsolaki, 2019). The patients were selected according to the Petersen criteria. All of the included patients had a diagnosis of multiple-domain aMCI subtype, an impairment in memory and at least one other neuropsychological domain was present. More specifically, both an episodic memory impairment (examined with California verbal learning test) and a deficit in at least one other cognitive domain, such as executive function (trail making part B), language (Boston Naming Test, verbal fluency task), or visuospatial skills (clock drawing test) were present.

The group of aMCI patients was examined first and then the healthy controls were recruited. The two groups were matched on demographic variables such as age, sex, and education levels. Both groups had the same experience with financial matters based on prior employment and other activities and had ranked themselves as having similar incomes. Both groups had similar history based on their self-reports on vascular risk factors and were all right-handed (for a brief discussion of the importance of controlling these two variables given their influence of financial capacity, see Giannouli and Tsolaki, 2022 and Giannouli and Milienos, 2024). The pool of healthy controls as well as the patients came from a larger prior study in northern Greece in which a group of volunteers with diverse demographics was formed (Giannouli et al., 2018). Participants were recruited from local memory clinics and seniors’ centers, with the following applied exclusion criteria: participants aged <65 years old, with a current or past diagnosis of a physical and/or psychiatric illness, having problems with vision and/or hearing, and not being a native Greek speaker.

All participants were tested with the same neuropsychological tests (for the patients’ detailed neuropsychological profile, see Giannouli and Tsolaki, 2019). The Rey auditory verbal learning test (RAVLT-immediate, delayed and recall conditions) was administered for the evaluation of verbal memory. The clock drawing test (CDT-immediate drawing and copy) measured spatial dysfunction and neglect. The trail-making test (TMT) parts A and B assessed visual attention and task switching. The Boston Naming Test (BNT) was used for measuring naming-word retrieval. The digit span memory test forward measures verbal short term memory and the digit span memory test backward measures executive functioning. Finally, the verbal fluency task (letter fluency and category fluency) were administered in order to assess phonemic and semantic verbal fluency.

After completing the neuropsychological assessment, all participants responded to Lusardi’s Big Three Questions which were used for assessing financial literacy, and more specifically, for assessing three financial constructs: knowledge of interest rates, inflation, and risk diversification (Lusardi and Mitchell, 2008). Although this set of three questions has been found to have a low Cronbach’s alpha in other countries, and in this sample Cronbach’s alpha was *α* = 0.416, it presents with high test–retest correlations at 6 months for this sample (*r* = 0.947, *p* < 0.001). These three questions are commonly accepted as the optimal measure for financial literacy measurement as they are designed to allow comparisons across groups, there are just three questions, and they are easy to use. In this study the three items were used as a total score (sum) as well as separate unique items-questions in the analyses.

Magnetic resonance imaging (MRI) acquisition was performed separately, and Statistical Parametric Mapping (SPM 12) was used only for post-processing and volumetric analysis. The automated anatomical labeling (AAL) atlas was used to extract regional volumes, and more specifically, for the volumetric assessment of white matter, gray matter, cerebrospinal fluid, and 16 additional brain areas (right angular gyrus, left angular gyrus, right amygdala, left amygdala, right precuneus, left precuneus, right hippocampus, left hippocampus, right parahippocampal gyrus, left parahippocampal gyrus, right thalamus, left thalamus, right medial superior frontal cortex, left medial superior frontal cortex, right medial frontal cortex, and left medial frontal cortex). Intracranial volume in this study was corrected for by dividing regional brain volumes by the total intracranial volume to create a ratio, normalizing for head size and accounting for overall brain atrophy. All scans were straight–non oblique, while the Human CORE Scan Protocol was followed (no adjustments were made to this protocol) (Giannouli and Tsolaki, 2023). The choice of including in measurements and analyses the above brain regions was made as Alzheimer’s disease is consistently associated with diminished volumes in regions of the default mode network (DMN), (namely posterior cingulate cortex (PCC)/precuneus, medial prefrontal cortex, hippocampus, medial temporal lobes, and inferior parietal lobules) as well as reduced functional connectivity and metabolism within this network (Suárez-Pellicini and McDonough, 2025), therefore justifying the interest in the selection of these brain areas. The study was conducted in accordance with the Declaration of Helsinki. Approval was obtained from the IRB committee of the university and informed consent was obtained from all participants.

## Results

For this research bootstrapped calculations involved sampling with replacement (1,000 samples) from the original data. The calculations were performed in R.

The Mann–Whitney *U* test, which is the non-parametric equivalent of the independent samples *t*-test, was used for comparing aMCI patients’ financial literacy (total score of three questions) with that of the healthy controls. There was a statistically significant difference between the two groups, following a bootstrapping approach (1,000 samples, bias-corrected and accelerated confidence interval, BCa) which was used to construct a 95% confidence interval for the difference between the medians. Results indicated that healthy older adults had higher financial literacy (Median_controls_ = 2.00, IQR: 1.00–2.00) than aMCI patients (Median_MCI_ = 1.00, IQR: 0.00–1.00), *U* = 61.00, *p* = 0.027, 95% CI (0.074, 0.723) (see Table 1).

**Table 1:** Means and standard deviations for the demographics of the two matched groups.

Variables	aMCI patients	Controls	*p*
Age	70.0 (8.31)	69.80 (8.06)	0.947
Education—in years	10.00 (3.70)	9.86 (3.06)	0.915
MMSE	28.06 (1.62)	29.66 (0.48)	<0.001
GDS	0.60 (0.91)	0.53 (0.64)	0.818

Spearman’s rho correlations were also applied for the aMCI group between their brain volumes, neuropsychological test performance and financial literacy-total number of questions answered correctly (raw scores) as well as for each individual item response. Spearman’s correlations revealed that a number of correlations exist specifically for aMCI patients data (see Table 2 with means and SDs for brain volumes and neuropsychological tests for the aMCI group).

**Table 2:** Descriptive statistics for brain volumes and neuropsychological test performance for the aMCI patients.

Brain volumes and neuropsychological tests	Mean	SD	Minimum	Maximum
Gray matter	604.400	65.889	470.000	758.000
White matter	468.333	40.771	367.000	543.000
Cerebrospinal fluid	244.600	31.382	198.000	305.000
Left amygdala	1.078	0.161	0.782	1.348
Right amygdala	1.069	0.137	0.805	1.322
Left angular gyrus	5.850	1.204	4.195	8.599
Right angular gyrus	7.200	1.204	5.455	9.393
Left frontal medial cortex	1.332	0.200	0.989	1.640
Right frontal medial cortex	1.113	0.202	0.642	1.428
Left superior medial frontal gyrus	4.233	0.586	3.339	5.467
Right superior medial frontal gyrus	4.389	0.563	3.623	5.577
Left hippocampus	3.762	0.290	3.176	4.098
Right hippocampus	4.148	0.306	3.528	4.633
Left precuneus	7.828	1.100	4.931	9.985
Right precuneus	8.483	1.126	5.914	10.759
Left parahippocampal gyrus	2.444	0.210	2.067	2.860
Right parahippocampal gyrus	2.255	0.217	1.883	2.709
Left thalamus	4.526	0.606	3.234	5.642
Right thalamus	4.452	0.717	3.393	6.349
Mini Mental State Examination	28.867	1.432	25.000	30.000
Rey auditory verbal learning test immediate recall	39.933	11.202	19.000	58.000
Rey auditory verbal learning test delayed recall	6.733	3.474	1.000	12.000
Rey auditory verbal learning test recognition	13.667	1.345	12.000	15.000
Clock drawing	4.600	0.737	3.000	5.000
Clock copy	4.733	0.458	4.000	5.000
Trail making part A (in seconds)	70.933	42.894	23.000	175.000
Trail making part B (in seconds)	194.333	112.334	62.000	400.000
Boston Naming Test	21.067	5.365	12.000	28.000
Digit span forward	6.000	0.926	5.000	8.000
Digit span backward	3.933	1.580	2.000	6.000
Verbal fluency task-phonemic/Letter fluency	30.667	9.108	18.000	44.000
Verbal fluency task-semantic/Category fluency	42.067	11.659	23.000	65.000

For example, financial literacy (total score of three questions) correlates with a number of brain volumes (after applying Bonferroni corrections for multiple comparisons) such as white matter [rho = 0.624, 95% CI (0.03, 0.95), *p* = 0.0013], gray matter [rho = 0.800, 95% CI (0.44, 0.95), *p* < 0.001], left amygdala [rho = 0.850, (0.58, 0.95), *p* < 0.001], and right frontal medial cortex [rho = 0.821, (0.47, 0.96), *p* < 0.001].

More specifically, for the first question (measuring compound interest literacy) statistically significant correlations were found between responses and gray matter volume [rho = 0.772, (0.43, 0.92), *p* < 0.001], right amygdala [rho = 0.850, (0.59, 0.954), *p* < 0.001], and left amygdala [rho = 0.756, (0.39, 0.91), *p* = 0.001]. For the second question (measuring inflation literacy) and for the third question (measuring risk diversification) no statistically significant correlations were found among cognitive and brain volume variables. It is of interest to mention that neither age nor education correlated with the Big Three Questions.

Among the cognitive tests, correlations were only found between financial literacy (total score) and the TMT-A completion time recorded in seconds [rho = −0.657, (−0.87, −0.21), *p* = 0.0008] and the TMT-B completion time recorded in seconds [rho = −0.634, (−0.86, −0.18), *p* = 0.0011].

## Discussion

This preliminary study demonstrates that, in the relatively understudied Greek cultural context, financial literacy responses differ between aMCI patients and healthy older adults, even when controlling for sex, education, socio-economic status, and prior financial experience. Although general literacy (years of formal education) and depressive symptomatology (as measured by the GDS) did not influence performance on the Big Three Questions (Giannouli and Tsolaki, 2021a; Giannouli and Tsolaki, 2021b; Giannouli et al., 2022), healthy older adults still outperformed aMCI patients. Notably, both groups were living independently, without hospitalization or supervision, highlighting the potential financial vulnerability and risk of exploitation among individuals with aMCI.

Additionally, specific brain volumes and cognitive measures, assessed using standard neuropsychological tests, were associated with financial literacy in this sample. Beyond general indicators of brain health, such as white and gray matter—which have been reported in prior studies from the U.S. (Han et al., 2016)—volumes of the medial frontal cortex and bilateral amygdalae, regions implicated in executive functioning, decision-making, and emotional regulation, were related to performance on financial literacy tasks. These findings suggest that combining structural brain imaging with cognitive assessments may help identify individuals at risk and could inform clinical evaluations or interventions in Greece.

Interestingly, the role of cognitive tests such as the TMT parts A and B as an important test to consider for financial literacy correlates is also highlighted. Although TMT parts A and B are key neuropsychological tests measuring cognitive functions such as processing speed (in part A) and mental flexibility/executive functions (in part B), they are vital not only for detecting cognitive impairment in old age, but also for detecting financial capacity deficits (Giannouli, 2024).

There were some limitations of the current study which need to be addressed in future studies, such as the criticism regarding the use of the Big Three Questions for the assessment of financial literacy (given the restricted scope of the financial literacy measure and the low internal consistency in this sample; Cronbach’s *α* = 0.416), as well the methodological limitations linked to the small sample size of participants and the potential recruitment bias (patients recruited first and controls from a prior study). Although the small sample size may limit the interpretability and potential impact of the findings (with concerns regarding statistical power, robustness of correlations, and generalizability, particularly given the large number of brain regions analyzed), this study has potential value as an exploratory contribution to an understudied research area. Given the scarcity of research in Greece on this topic and despite the limitations this study presents several strengths as a detailed neuropsychological and physical assessment was provided and the two groups were matched on a one-to-one basis. In future work, an attempt will be made to address these issues, but these preliminary findings may still have utility in the assessment of older patients and by serving as a basis for future research in larger samples. Given the strong correlation between financial literacy and retirement planning and savings and wealth accumulation across countries (Lusardi et al., 2014), these findings provide variables that healthcare professionals and policymakers should take into consideration for the group of older people who have a diagnosis of neurocognitive disorder.

## Data Availability

The raw data supporting the conclusions of this article will be made available by the authors, without undue reservation.
